# AI-supported qualitative analysis of free-text responses on home care burden and support needs in Saxony

**DOI:** 10.1038/s41598-026-46989-7

**Published:** 2026-04-02

**Authors:** Elisabeth Rau, Silke Geithner, Tom Schaal

**Affiliations:** 1 Faculty of Health and Healthcare Sciences, University of Applied Sciences Zwickau, Kornmarkt 1, 08056 Zwickau, Germany; 2 University of Applied Sciences for Social Work, Education and Care, Dresden, Germany

**Keywords:** People in need of care, Home care challenges, Informal caregivers, AI-assisted qualitative analysis, Free-text survey responses, Health care, Health humanities, Medical humanities

## Abstract

The number of people in Germany requiring care has risen steadily, increasing the importance of informal care. This form of care is often associated with considerable psychological and physical strain. The aim of this study is to systematically categorize and qualitatively analyze the free-text responses from a survey on home care using an artificial intelligence-based approach in order to identify key challenges and support needs in home care from the perspective of informal caregivers and non-caregiving relatives. The study used data from a 2019 survey on home care in Saxony. Free-text responses were categorized and analyzed using GPT-4 Turbo within a hybrid human-AI workflow. All AI outputs were subsequently validated and corrected by researchers. Respondents reported substantial financial burdens for both care recipients and informal caregivers. They also highlighted structural barriers to accessing services and insufficient support from the care system. Improving home care requires structural measures, including the expansion of low-threshold counseling services, more flexible leave regulations, stronger financial security for informal caregivers, and the sustainable strengthening of care infrastructures. Given an AI error rate of 36.45%, the study emphasizes the need for human post-processing to ensure analytical accuracy.

**Supplementary Information:**

The online version contains supplementary material available at 10.1038/s41598-026-46989-7.

## Introduction

In 2023, there were just under 5.7 million people living in Germany who needed care. This represents an increase of around 730,000 people in need of care compared to 2021^1^. According to forecasts, the number of people in need of care is expected to rise further in the coming years^2^. At the same time, a growing gap in professional care provision is emerging. According to forecasts, the demand for nursing staff will rise to around 2.15 million by 2049. In the status quo scenario, which only takes demographic changes into account, a shortage of up to 690,000 nursing staff is predicted^3^. The shortage of skilled workers in the care sector has far-reaching consequences for both nursing staff and those in need of care. Nursing staff are confronted with increasingly challenging working conditions. The shortage of skilled workers in the care sector also leads to limited access to needs-based care services for care recipients. There are deficits in the availability of care offers, particularly in structurally weak and rural regions^4^. The rising costs of accommodation in inpatient care facilities also pose a growing challenge for many people in need of care^5^.

Against the backdrop of the predicted shortage of skilled workers, informal caregivers are becoming increasingly important. In 2023, 4.89 million people in need of care were cared for at home, with the majority of this care provided by relatives^3^. This non-professional form of support is referred to as informal care and includes the care and support of people in need of care by close persons such as family members, friends, or neighbors who have neither professional nursing training nor a formal employment relationship in the care sector^6^.

The majority of informal caregivers in Germany are women. People between the ages of 50 and 70, in particular, often take on caregiving tasks within the family^6^. The study by Schwinger and Zok (2024) showed that the amount of time spent on home care has increased significantly in recent years. In 2023, informal caregivers spent an average of 49 h per week on caregiving activities. This high time commitment has a particular impact on the employment of primary caregivers of working age^7^. In addition, this type of care is often physically demanding and emotionally stressful, especially when there are additional professional and family commitments^8^.

In the course of recognizing the burdens of informal carers, legal measures have been implemented that aim to structurally improve the support conditions. These include financial benefits and care services that provide relief^9^. In addition, from July 2025, informal caregivers will be able to take up to ten days of paid leave per year to organize care^10^. Furthermore, a reform of long-term care is currently being pursued, which will, among other things, make the existing exemption rules more flexible and further develop the pension insurance coverage for family caregivers.

Despite legislative improvements, a large portion of the available support services is not utilized to a corresponding extent. The study by Rueffer et al. (2020) indicates that the utilization of benefits provided by the long-term care insurance system by informal caregivers largely depends on the intensity and duration of care required, as determined by the care level. Monetary benefits such as care allowances are more frequently used when caregiving demands are high, whereas relief services like respite care are accessed less often^11^. Key reasons for the comparatively low uptake include insufficient information about entitlement to benefits and the complex structure of the care system, which makes navigation and application processes more difficult. Additionally, bureaucratic barriers and burdensome application procedures present a significant access hurdle, particularly for older individuals and those with health limitations^12^.

In view of the challenges outlined above, a deeper understanding of the perspectives of people in need of care and informal caregivers is essential in order to further develop targeted support measures. In particular, qualitative findings on individual burdens of care, needs, and suggestions for improvement can provide valuable information for the needs-based design of care structures.

Against this background, this study aims to systematically categorize and qualitatively analyze the free-text responses collected in the home care survey in Saxony (a federal state in Germany) using an artificial intelligence–based approach, in order to identify key challenges, needs and suggestions for improvement from the perspective of informal caregivers and non-caregiving relatives, and to make these findings usable in health and care policy practice. Additionally, the results will inform the development of practical implications for further care support service development.

## Methods

This empirical study was based on a survey on home care in Saxony conducted in 2019. The aim of the study was to analyze the situation of informal caregivers in Saxon households. The survey was designed as a cross-sectional study and was conducted using a questionnaire aimed at both informal caregivers and non-caregiving relatives^11,13–16^. The evaluation is a secondary analysis of a data set that was not collected independently and includes a qualitative evaluation of the free-text responses performed with the help of AI^17^. The questionnaire and the sampling frame were not originally developed with regard to a secondary free-text analysis.

### Sample

The sample was recruited actively through the registration offices of the Free State of Saxony. A total of 62 registration offices provided resident data for 26,576 individuals. After methodological stratification of the data, considering the population of the cities and municipalities, 24,018 registration data were available. The study included residents of Saxony aged between 40 and 85 (target population). Receipt of statutory care services by a person potentially in need of care was not an inclusion or exclusion criterion^11^.

### Data collection and survey instrument

Recruitment took place in two phases. The first phase began on November 5, 2019, with the distribution of postcards. A second phase started on December 12, 2019. In this context, potential participants were invited to voluntarily participate in a web-based questionnaire entitled “Survey on Home care in Saxony.” Potential participants who preferred not to participate online had the option of requesting a paper-based questionnaire. The questionnaire was partially standardized and validated. It consisted of 67 items that mainly related to the participants’ home care situation, their use of long-term care insurance benefits, and their well-being in various areas of life. At the end of the questionnaire, participants were provided with an optional open-ended question inviting additional comments. Completion of this field was voluntary. An online pretest was conducted before data collection began. To describe the sample, background information on the characteristics of the participants, such as gender, age, marital status, and educational level, was collected. In addition, the variable “informal care” was used to determine whether the respondent provided care and support for a person close to them^11^.

All individuals in the sample (*N* = 24,018) were invited to participate. 1,301 individuals took part in the survey online. 415 individuals completed the paper version and returned it. Both types of survey were combined to form a representative data set (gross response rate = 1,716). The gross response rate was 7.15%. 201 invitation cards could not be delivered. At least eight individuals refused to participate or were unable to participate. 16 questionnaires were excluded (net response rate = 1,700) because the participants did not meet the age criterion. The net response rate was 7.14% (*n* = 23,793)^11^.

### Data analysis

As part of the data analysis, the free-text responses from Question Box 68 of the survey on the care situation in Saxony, available online via Reference 15, were structured and analyzed using the reflexive thematic analysis method developed by Braun and Clarke. This approach was chosen due to its theoretical and practical flexibility and broad applicability. As the phases of reflexive thematic analysis are not necessarily linear but recursive in nature, their sequence was adapted in this study to the requirements of the AI-supported procedure. The analysis was carried out using large language models (LLMs), which provided algorithmic support for phases one and two of the thematic analysis (familiarization and coding)^18^. The analysis was conducted with ChatGPT (GPT-4 Turbo model, OpenAI, November 2023 release; execution period: April 15–22, 2025), which is based on the Generative Pre-trained Transformer (GPT) architecture. The model supports multimodal inputs and offers an extended context window of up to 128,000 tokens^19^. A neutral system instruction guided the classification task (“You are an assistant that classifies free-text responses from caregivers and non-caregivers according to predefined thematic categories in the context of public health research.”). Default model settings were used, including temperature, token limits, and other parameters, without further modification. The standard context window was sufficient for all text segments, and no additional chunking was required. As the free-text responses were written in German, all category definitions, prompts, and model interactions were likewise formulated in German to ensure linguistic consistency. GPT-4 Turbo was selected for its strong performance in complex text classification, high context capacity, stable interface behavior, and proven multilingual capabilities. Compared to open-weight alternatives, it offered a favorable combination of computational efficiency, language coverage, and reproducibility within the ChatGPT environment. Its contextual understanding of natural language and ability to capture semantically related expressions facilitated the efficient structuring of heterogeneous free-text responses, whereas conventional topic modeling or rule-based tools were less suitable for this purpose.

The analyzed free-text data came from a publicly accessible, completely anonymized data set. Before use, it was verified that no directly identifiable personal information was included; potentially identifying information was redacted. The cleaned data were uploaded to ChatGPT in accordance with institutional guidelines on the use of AI-based analysis tools and were legally and ethically permissible due to the public and anonymized nature of the data. To protect data privacy, no case numbers or assignment codes were transmitted, so that no conclusions could be drawn about individual persons.

After adjustment for noncompliance with the age range, the net return was 1,700 out of 1,716. Of the 1,700 individuals, 334 (19.6% of the net sample) provided information in the free-text response option, with 332 ultimately included in the analysis.

**Fig. 1:**
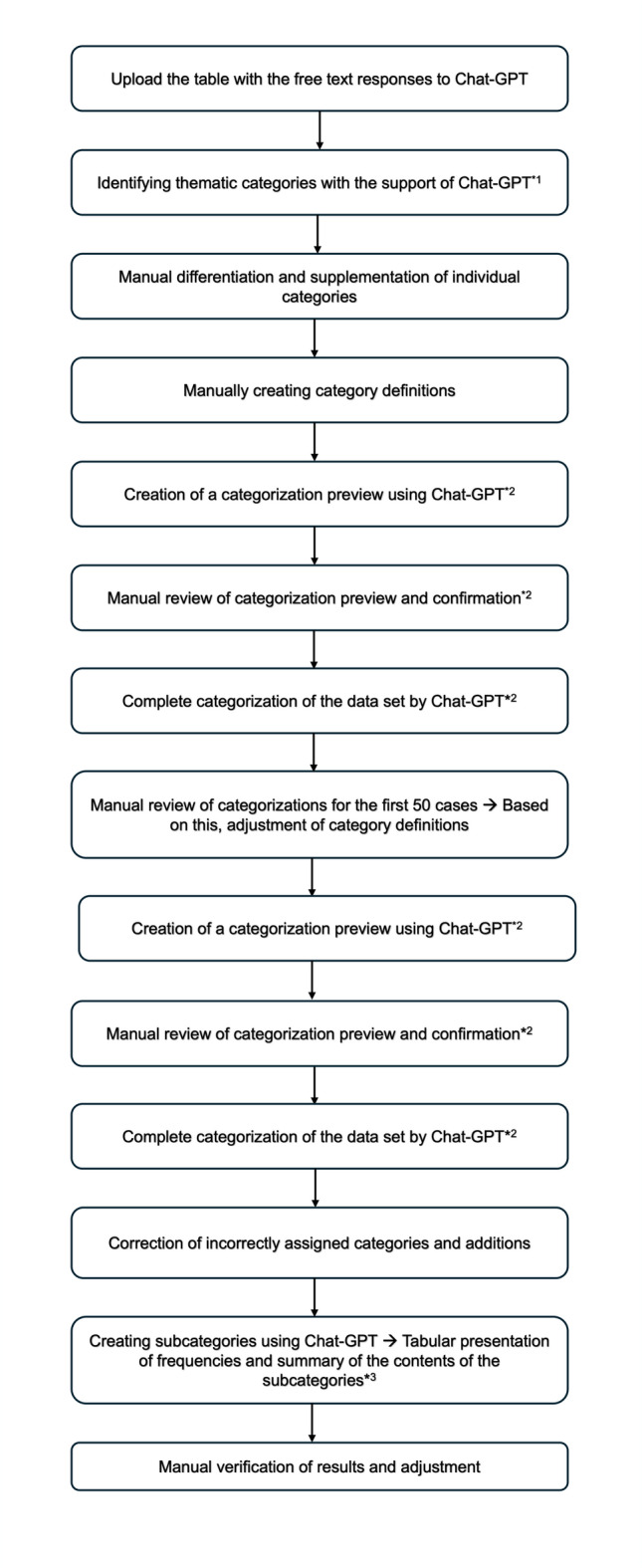
Workflow of AI-supported qualitative analysis of free-text responses. The original prompts used in each step of the workflow, as well as their English translations, are provided in the Supplementary Material.

First, the free-text responses were extracted from the overall data set and transferred to a spreadsheet (Microsoft Excel), which was then uploaded to ChatGPT. An overview of the individual steps is shown in Fig. 1. The input prompts used for ChatGPT in the individual steps are shown in the supplementary materials in notes 1a and 1b. In the next step, ChatGPT was instructed to use this data to identify thematic categories contained in the responses. In addition, the categories “Comments on the questionnaire” and “No information provided or content cannot be assigned” were added.

A definition was created for each category, which was illustrated with examples from the data set. The AI was then tasked with assigning each response to one of the previously defined categories. The category that best reflects the content of the response should be assigned. In addition, the AI’s assignment should be validated in a preliminary step. To this end, two responses from the data set were assigned to each of the defined categories as examples, and the assignment was justified. This preview was checked manually to assess the reliability of the categorizations. Once all categories had been assigned correctly, ChatGPT was instructed to categorize the entire data set and present the results in a table. An overview of the individual correction rounds is provided in Supplementary Fig. 1.

A manual review of the categorizations was then carried out for the first 50 cases. Particular attention was paid to the linguistic features that led to incorrect assignments. The findings were used to further specify and adjust the category definitions. The steps described above were then repeated.

Finally, the final table output by ChatGPT was checked in its entirety. Responses with incorrect categorizations were marked in red, while multiple assignments (i.e., categories that were relevant in terms of content but could not be clearly assigned) were marked in yellow. In addition, an extra column was added in which incorrect assignments were corrected or additional suitable categories were added.

The next step was to further differentiate the individual categories. To do this, a table with the corresponding answers was first created for each of the main categories. These tables were uploaded separately to ChatGPT. ChatGPT was then instructed to identify subcategories for selected main categories based on the content focus of the respective answers. In addition, the frequency of mentions for individual subcategories was to be recorded quantitatively and the content of the respective responses summarized. No subcategories were created for the main categories of mental stress and emotional overload, time pressure, comments on the questionnaire, no or little experience with care, and no information provided or content not assignable. Analysis of the content of the corresponding text segments showed that the responses within these categories were very similar and referred exclusively to the generic term. Further differentiation in terms of content therefore appeared neither theoretically useful nor empirically justified. The low variance in the statements and the semantic homogeneity within these categories did not allow for a clear subcategorization. Finally, all of the results generated by ChatGPT were manually reviewed and validated.

## Results

Of the 1,700 participants, a total of 334 provided a response using the free text option. The average year of birth for those who did not provide a response was 1957.65 (SD = 11.8), while those who did provide a response were born slightly earlier on average (M = 1954.7; SD = 12.0). A total of 1,696 people provided information about their gender. Among the participants who did not provide any information in the free text option, 719 (52.7%) were female and 639 (46.8%) were male; six people (0.4%) identified themselves as “diverse.” In the group of participants who wrote an open-ended response, 186 (56.0%) were female and 145 (43.7%) were male; one person (0.3%) identified as “diverse.”

**Fig. 2:**
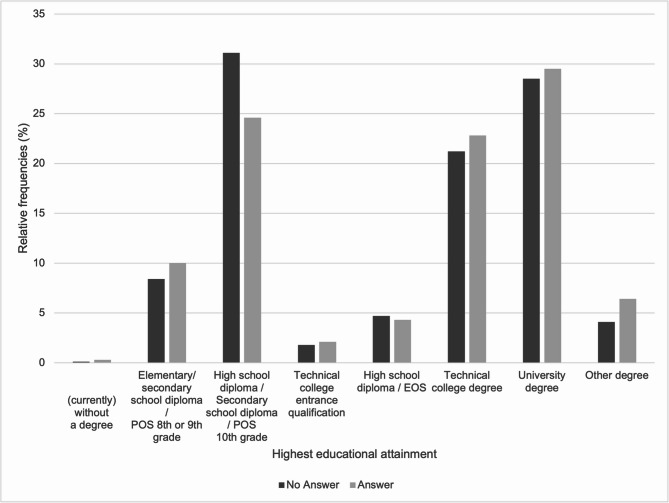
Distribution of participants by highest educational attainment and open-text response status (*N* = 1,695; n (answer) = 329 1.5% missing values; n (no answer) = 1,366 0.0% missing values).

The distribution of participants by highest educational attainment and open-text response status is summarized in Fig. 2. The largest groups held a secondary school diploma (*n* = 507; 29.9%) or a university degree (*n* = 487; 28.7%). Of the individuals with a secondary school diploma (*n* = 507; 29.9% of the sample), 81 (16.0%) provided a free-text response, while 426 (84.0%) did not. Among participants with an university degree (*n* = 487; 28.7%), 97 (19.9%) wrote a free-text response, while 390 (80.1%) did not provide a free-text response.

**Fig. 3:**
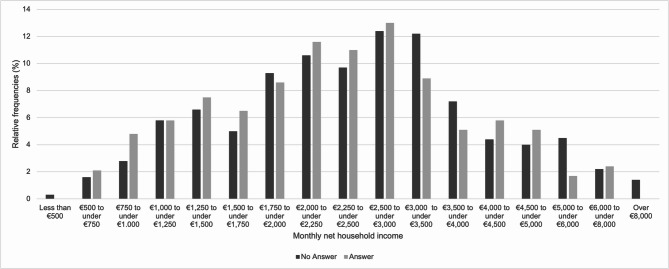
Distribution of participants by monthly net household income and open-text response status (*N* = 1,497; n (answer) = 296 11.4% missing values; n (no answer) = 1,201 12.1% missing values).

Figure 3 summarizes the distribution of participants by monthly net household income and open-text response status. Most respondents reported an income between €1,750 and €3,500.

**Fig. 4:**
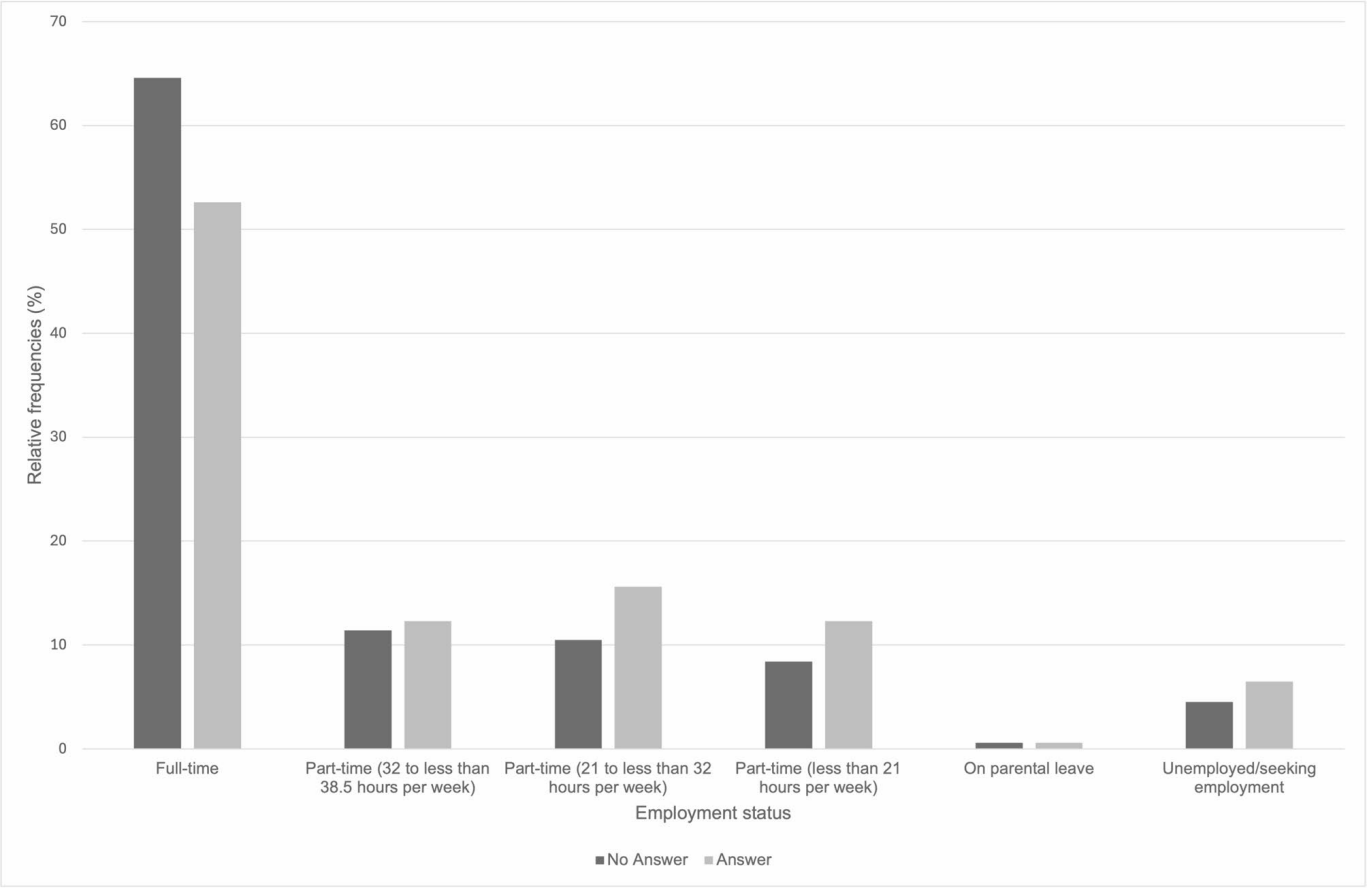
Distribution of participants by employment status and open-text response status (*N* = 880; n (answer) = 154 53.9% missing values; n (no answer) = 726 46.9% missing values).

Figure 4 displays the distribution of participants by employment status and open-text response status. The majority were employed full-time (*n* = 550; 62.5%). Among the participants who were employed full-time (*n* = 550; 62.5% of the sample), 81 people (14.7%) provided a free-text response, while 469 people (85.3%) did not provide any free-text information.

The question of whether participants had provided informal care within the last 12 months was answered by 1,694 people. Of those who provided information in the free text option, 93 people (28.2%) stated that they had provided informal care within the last 12 months, while 237 (71.8%) did not. Of those who did not use the free text option, 218 (16.0%) stated that they had provided informal care within the last 12 months, while 1,146 (84.0%) did not.

Of the 334 free-text responses, two responses were excluded because these fields were left unfilled. A total of 332 responses were included in the analysis. Two responses were assigned to the category “no or little experience in care.” 25 responses were assigned to the category “comments on the questionnaire”, while 57 did not contain any information on home care or could not be assigned to any category. The individual main and subcategories and a selected excerpt of the corresponding frequencies are shown in Table 1. The complete frequency distribution is provided in the Supplementary Table S1.

**Table 1:** Selected frequencies of mentions by subcategory.

Main category	Subcategories	Number of mentions (*n*)	Number of mentions (%)
Personal wishes and specific suggestions for improvement	Personal wishes		
	Care at home instead of in a nursing home, remaining independent for as long as possible	15	4.5
	No burden on children/family due to care	6	1.8
	Specific suggestions for improvement		
	Better pay and more staff in nursing care	20	6.0
	Improve the financial situation of home care	16	4.8
Application process	High bureaucratic effort	10	3.0
	Incorrect classification by medical services	6	1.8
Financial burden	Direct financial burden due to care costs	6	1.8
	Inadequate support from the system/lack of relief	4	1.2
Experience as a informal caregiver or with nursing homes or professional care services	Experience as a informal caregiver		
	High stress/exhaustion	15	4.5
	Lack of support/relief	11	3.3
	Experience with nursing homes/outpatient care services (subcategories)		
	Staff shortages/time pressure	18	5.4
	Costs and financing problems	13	3.9
	Quality of care varies greatly	10	3.0
	Impersonal and passive home environment	8	2.4
Information on the care situation (description of individual case)	Care of relatives (current or completed)		
	Care of parents	20	6.0
	Care of husband/wife	8	2.4
	Own need for care		
	In need of care with care level	10	3.0
	Serious illness without care level	6	1.8
	Deceased person in need of care		
	Care ended due to death	15	4.5

Percentages refer to the total number of free-text responses (*N* = 332).

### Personal wishes and specific suggestions for improvement

A total of 79 people expressed personal wishes and specific suggestions for improvement in the area of care in their responses. With regard to specific suggestions for improving the care system, most of those surveyed mentioned better pay and more staff in the care sector. This was justified in particular by the fact that the current workload for nursing staff is perceived as too high, which in turn has a negative impact on the quality of care.

In addition, 16 people stated that financial support for home care provided by relatives needs to be expanded. Measures such as the introduction of wage replacement benefits during leave, tax advantages, or pension points were also mentioned. Five people would also like to see better compatibility between caring for relatives and their own jobs.

Nine respondents said that they would appreciate a reduction in bureaucracy. Suggestions in this regard include the automatic provision of relevant information and simplified application procedures. Five of those surveyed criticize the rigid structure of care services. They would like to see individual adaptation to actual needs, for example in the case of aids such as care gloves or travel costs. Four people stated that care in rural areas needs to be improved. In this context, the current infrastructure in terms of local public transport, medical care, and shopping facilities was particularly criticized. Six people called for the expansion and improvement of information options for care recipients and their relatives. They should be provided with specific information about their rights, entitlements, and the procedure for applying for assistance. In the respondents’ view, this should be done by family doctors or health insurance companies before the need for care arises.

### Application process

A total of 28 people commented on aspects of the application process in their responses. In this context, ten of the people surveyed commented on the high level of bureaucracy involved in applying for benefits. They criticized the fact that many documents must be submitted and extensive forms have to be filled out. The application process is described as time-consuming and burdensome. In addition, there is criticism that many procedures continue to be paper based despite the availability of digital options.

Furthermore, four people stated that the wording in applications for approval was often unclear or ambiguous. It was often not clear what exactly was meant or how certain questions should be answered. This leads to uncertainty and makes it difficult to submit a complete and correct application for approval.

Four people criticized the sometimes confusing and poorly coordinated communication with the relevant authorities in the care and support system. Examples included misunderstandings regarding the requirements and procedures for applying for or billing individual services, which led to uncertainty and, in some cases, delays in receiving benefits.

In addition, five people criticized the system for lacking transparency in the context of the application process. It is often unclear which department is responsible for which application, which services can be combined, and which requirements apply in each case.

### Experiences as an informal caregiver or with nursing homes or professional care services

A total of 66 people commented on their experiences as an informal caregiver or with nursing homes or professional care services in their responses.

In the context of their experience as an informal caregiver, 15 people mentioned the high level of strain caused by caregiving. The strain described was both physical and psychological.

Eleven of the respondents stated that they felt inadequately supported and often left to their own devices when caring for their relatives. Criticism was directed at the lack of short-term respite services, the lack of replacement options in the home environment, and the insufficient compatibility of work and care responsibilities.

Nine people stated that information and counseling services in the care context were often perceived as inadequate, difficult to access, or not tailored to the target group. Particularly in new care situations, respondents reported difficulties in obtaining an overview of existing support services within a short period of time.

Based on their experience with nursing homes and outpatient care services, 18 people stated that the existing staff shortage in nursing homes and outpatient care services was a key problem. The resulting overload on existing staff had far-reaching effects on the quality of care, the well-being of those in need of care, and the working conditions of caregivers. Nursing staff would often be under time pressure and unable to respond to the individual needs of those requiring care due to staff shortages. This would lead to basic care in which personal attention, communication, and social interaction would hardly be possible.

13 people stated that the costs of inpatient care or outpatient care services would exceed the financial means of those affected. Statutory pension benefits are perceived as inadequate, so that private savings or financial support from relatives often must be used.

Eight people describe nursing homes as facilities where the individual needs and personal realities of residents are not adequately considered. In particular, they criticize a lack of personal attention, empathy, and activating care. Accommodation is primarily aimed at supervision and care. People in need of care who are no longer able to express themselves actively are particularly disadvantaged.

### Financial burden

17 people mentioned a financial burden caused by their care situation in their responses. Six of the people surveyed mentioned direct financial burdens caused by care costs. For example, they said that care costs exceeded the pension income of the people affected. This applies not only to general co-payments for inpatient care facilities, but also to additional expenses for other services such as personal hygiene, which are not fully covered by long-term care insurance. In addition, the increase in the cost of a place in a nursing home was mentioned. Relatives must bear a large part of the nursing home costs themselves, especially if they have several parents in need of care.

Structural deficits in terms of job security after the end of care periods were also pointed out. Respondents stated that they faced considerable disadvantages in their employment history, for example as a result of interrupted employment, lack of pension entitlements, or difficulties in re-entering the labor market. In addition, four people mentioned that the compatibility of care and gainful employment is associated with considerable restrictions. For example, care obligations sometimes lead to job loss or require a significant reduction in working hours, which has a direct impact on the income and long-term financial security of those affected. Particularly problematic in this context is the fact that voluntary or informal care work is often not recognized for pension purposes. People who take on care tasks as part of mini jobs generally acquire little or no pension entitlements.

### Mental stress and emotional overload

Ten people mentioned significant mental stress caused by a care situation in their responses. Coordinating care is particularly challenging when it must be done alongside a job. In this case, respondents reported a lack of opportunities for rest and recovery, which leads to emotional exhaustion and social isolation. In addition, respondents reported feeling overwhelmed when faced with new care situations. In this context, fears of making the wrong decisions play a particularly important role.

### Time pressure

Three people mentioned the time pressure involved in caregiving in their responses. The considerable coordination effort required, even with support from health insurance companies, general practitioners, and care services, was particularly challenging. This would lead to considerable time pressure in conjunction with caregiving and full-time employment. According to the respondents, caregiving tasks severely restrict everyday life. For example, there would be hardly any time for personal appointments and commitments. Short-term caregiving tasks would also frequently interrupt or prevent other everyday activities.

### Limitations

This study, grounded in Braun and Clarke’s flexible methodology, offers a robust framework for data-oriented analysis and interpretation of both manifest and latent meanings^18^. Nevertheless, the AI-supported approach revealed limitations, particularly with ambiguous statements. Among 332 free-text responses, 178 were correctly categorized, 121 were misclassified, necessitating corrections, and 33 required additional categories, highlighting AI’s contextual and multidimensional challenges. Errors often involved misinterpretation of situational context, reducing complexity to single categories, thus necessitating human recoding to ensure depth in qualitative findings. Despite ChatGPT-4-Turbo’s rapid processing capabilities, its limited nuanced language comprehension and contextual interpretation resulted in a 36.45% error rate in this specific classification task and dataset, underscoring the need for human expertise in validating AI-based categorizations. Importantly, a fully manual analysis of this dataset would have been feasible; the AI component primarily served to support efficiency and initial structuring rather than methodological necessity. Against this backdrop, while AI aids in pre-structuring data, its benefits in small text samples are limited, as human review remains critical, although it holds potential for scalable, time-sensitive analyses. Moreover, AI models prioritize frequent linguistic patterns, potentially overlooking rare but important signals, mitigated here by iterative human review. This highlights AI’s in qualitative research, contingent on critical, methodical human oversight, utilizing both human context sensitivity and machine computing power^20^. Furthermore, less than a fifth of the total sample provided free-text responses. While these comments enriched the quantitative findings by offering nuanced insights, they must be interpreted as illustrative accounts of individual experiences rather than representative of the overall study population, particularly given the selective participation and lack of comprehensive demographic information. Because respondents providing comments systematically differed from the overall sample in terms of age and care profiles, the identified themes predominantly capture the perspectives of a specific subgroup. This limited specificity and differentiation across minority groups adds context-specific limitations to the study’s interpretations. Furthermore, it was not possible to distinguish whether the comments were provided by informal caregivers or by other family members because the data were uploaded to the AI system without case identifiers. In addition, it was not possible to identify whether the respondents themselves received dependency-related benefits, which further limits the specificity of the interpretations. It was also not possible to conduct a differentiated analysis of specific minority groups within the scope of this study.

## Discussion

The aim of this study was to systematically categorize and qualitatively analyze the free-text responses collected in the survey on home care in Saxony using an artificial intelligence-based approach. The evaluation of the free-text responses provides insights into the perspectives of informal caregivers and non-caregiving relatives and illustrates the range of challenges, needs, and suggestions for improvement that arise from their experiences and individual care situations.

With regard to their experiences as informal caregivers, many respondents described high levels of stress associated with caregiving in their free-text responses. Respondents reported both mental and physical stress. They attributed this burden mainly to the simultaneous demands of caregiving, paid work, and family responsibilities. The time commitment required by the caregiving situation also plays a decisive role in this context. The study by Rothgang and Müller (2018) came to a similar conclusion. In this context, for example, 85% of respondents said they cared for the person in need of care on a daily basis, with half of these people stating that this activity took more than twelve hours a day. This intensive workload leads to an increased experience of burden for a large proportion of caregivers. Around 38% of participants report sleep deprivation, just under 30% feel trapped in their role, and around one in five find caregiving permanently exhausting. In addition, caregiving tasks have a negative impact on social relationships^21^.

Furthermore, the respondents in this sample often described existing support and relief services as insufficient. Four comments referred specifically to comparatively limited care provision in rural areas. Looking at the actual use of the support services offered in this context, their utilization falls significantly short of the possibilities available, despite the existing entitlement basis. Results from various surveys show that services such as day care and short-term care are used relatively little, even though the corresponding entitlement criteria are met^11,12^. The reasons for the comparatively low use of such services include a lack of financial resources among those affected and regional differences in the density of care provision^4^.

However, another reason for the relatively low take-up could also be the complexity of accessing the support system as perceived by some of the respondents. From the perspective of three respondents in this sample, the lack of systematic and timely information about available support services is a key problem. Informal caregivers often must obtain information on their own. There is a particular lack of understandable, accessible, and target-group-specific informational materials. Studies have shown that existing knowledge about care-related services is unevenly distributed across different population groups. Those with higher levels of education, better digital skills, or specific professional backgrounds, such as in healthcare, tend to have greater opportunities to acquire knowledge. Meanwhile, socially disadvantaged people are particularly underrepresented^22^.

In this context, many respondents in this sample mentioned structural barriers to accessing support services. For some of those affected, accessing services involves considerable bureaucratic effort. The relevant forms are also often difficult to understand. The study by Hielscher et al. (2017) shows that people with lower socioeconomic status, limited German language skills, and those with a personal or family migration background are particularly disadvantaged when it comes to applying for benefits. There is often a lack of targeted support or assistance in completing and submitting applications, which means that benefits are not claimed or are claimed only after delays^12^. Social insurance institutions and administrative bodies should therefore simplify bureaucratic procedures and make application processes more accessible - for example, by introducing simplified forms, digital submission options, and personalized assistance for vulnerable groups.

In addition, five respondents in the present study criticized a lack of transparency with regard to responsibilities and insufficient communication with the service providers. A study shows that the unclear distribution of responsibilities, frequent changes in contact persons, and a lack of support in applying for and using care services are perceived as key challenges. For example, around one-third of the primary caregivers surveyed reported problems in their contact with these service providers^21^.

Furthermore, the results of this study highlight aspects that have received limited attention in the existing literature. A small number of respondents in this sample emphasized that standardized service packages in the long-term care system often do not meet the specific requirements of home care, for example due to rigid regulations on the provision of consumables or the reimbursement of transportation costs. These comments suggest a perceived discrepancy between administrative regulations and everyday caregiving practice. Given the limited number of mentions, these observations should be interpreted as illustrative rather than representative signals.

Suggestions to improve the compatibility of care and employment included more flexible working time models and paid leave arrangements. These proposals were articulated in several comments and align with ongoing policy discussions. Legislative measures allowing up to ten days of paid leave per year for informal caregivers from 2025 onward have already been adopted^10^. With regard to the structure of care services, some respondents expressed a desire for greater flexibility, as existing benefits in kind are often not sufficiently tailored to the individual needs of the person requiring care and are therefore frequently not taken up.

In order to better support people in need of care and their relatives in complex care situations, there is therefore a need for comprehensive, easily accessible, and binding care advice. In this context, low-threshold support and counseling services should be systematically expanded. In line with the recommendations of the Hallek et al. (2024), regional contact points operated by municipalities could play a crucial role here by providing guidance, identifying individual needs, and referring people to appropriate services^4^. In order to ensure access to relevant information, particularly in rural areas or for people with limited mobility, digital information and advice formats should be strengthened. In addition, everyday support services, such as household services, day care, and short-term care, which should be flexible in their use, should be expanded. One aspect that has rarely been addressed in the literature to date is the explicit desire for proactive contact by institutions. Respondents suggested that health insurance companies and general practitioners should actively inform individuals about possible care entitlements at important biographical moments, such as when a chronic illness is diagnosed or when a certain age limit is reached, rather than relying on those affected to make contact themselves.

Financial strain was another theme raised in the qualitative responses. In the responses, 17 people commented on the financial burden of a care situation. This financial burden relates on the one hand to the care recipients themselves and on the other hand to their informal caregivers. The costs of care often exceed the financial means of those in need of care. In the inpatient sector in particular, the monthly costs for many of those affected significantly exceed the benefits provided by social care insurance. For example, a survey conducted in 2018 showed that around 44% of relatives provide regular financial support^21^.

The respondents also mentioned indirect financial burdens resulting from their role as informal caregivers. In this context, four respondents in this sample mentioned in particular interrupted employment, reduced pension entitlements, and difficulties in returning to the labor market. The limited compatibility of caregiving and work also leads to a financial burden, as caregiving responsibilities sometimes result in job loss or require a significant reduction in working hours. The results of a study show that more than half of informal caregivers who work part-time have reduced their working hours due to caregiving. In addition, almost one-third of those who are no longer employed stated that they had given up work completely due to caregiving responsibilities^7^. Women, who predominantly provide informal care, are particularly vulnerable to financial disadvantages due to loss of earnings and the resulting reduced pension entitlements, also in combination with other periods of family care^22^. Closely related to these findings is the desire expressed by respondents that home care should be better funded.

The challenges raised by respondents in the context of financial burdens can be linked to current challenges in the area of care financing, which require a reform of social care insurance and greater consideration of the financial situation of people in need of care and their relatives. Initial legislative measures have already been initiated, including an increase in care allowances and the consolidation of respite and short-term care into a flexible annual budget^10^. However, it is also necessary to improve social security options for informal caregivers. For example, existing regulations regarding pension entitlements, which are often negatively affected by periods of care, should be reviewed and subsequently adjusted.

With regard to their experiences with inpatient care facilities or outpatient care services, some respondents in this sample described a serious shortage of staff, which in turn leads to time pressure and a reduction in the quality of care. According to the respondents, these aspects are reflected, among other things, in the inadequate alignment of care provision with the needs of those requiring care and a general failure to take personal circumstances into account. Against this backdrop, the respondents repeatedly expressed the desire for better staffing levels in care facilities and appropriate remuneration for care staff. With regard to the staffing situation in the care sector described by the respondents, it is particularly evident in inpatient care facilities that insufficient staffing not only places a massive strain on the working conditions of nursing staff, but also has a negative impact on the quality of care. In this context, nursing staff report increasing workloads, overtime, a lack of time for conversations, and a lack of individual care. Under these conditions, care is often reduced to basic care, while social attention and rehabilitative measures are hardly possible. Studies show that inadequate staffing can lead to an increase in avoidable complications such as pressure ulcers, malnutrition, and dehydration. There is also an increased risk of hospital stays that could have been prevented with better care in nursing homes^23^.

Various approaches have been developed to address the shortage of skilled workers. According to Hallek et al. (2024), one focus should be on improving working conditions, for example through reliable duty rosters, adequate staffing levels, and the use of multi-professional teams. To provide short-term relief, structured and fair access to the labor market for international nursing staff is recommended^4^.

## Conclusion

The analysis of the free-text responses on home care in Saxony has shown that informal caregivers face a wide range of challenges. Clear areas for action can be derived from the experiences and needs of the respondents. Key recommendations include the expansion of low-threshold and comprehensible counseling and information services that specifically address educationally disadvantaged and marginalized groups. Furthermore, there is a need for better compatibility between care and work, for example through flexible working time models, as well as a structural upgrading of informal care through better social security, higher pension entitlements, and financial relief. Measures to improve working conditions and attract skilled workers should also be developed in the formal care sector.

This study provides valuable insights into the state of home care in Saxony, offering important implications for strengthening the role of informal caregivers. The findings serve as guidance for policymakers and health insurance companies, suggesting avenues to further alleviate both the burdens on caregiving family members and the challenges faced by the inpatient care sector. However, the free-text comments should be understood as illustrative accounts of specific experiences rather than representative of all informal caregivers in Saxony. Although the conclusions are drawn from data specific to Saxony, these findings should be interpreted within their regional context, and future research should include perspectives from other federal states to construct a more comprehensive picture of the care landscape in Germany.

It is also noteworthy that this publication represents one of the first published applications of GPT-4-supported qualitative analysis of large-scale survey free-text responses in the German care context. By aggregating individual feedback from a comprehensive dataset into core findings, this approach facilitated an efficient synthesis of the material, while a manual analysis would likewise have been feasible but considerably more resource-intensive.

## Supplementary Information

Below is the link to the electronic supplementary material.

Supplementary Material 1

Supplementary Material 2

Supplementary Material 3

Supplementary Material 4

## Data Availability

All data analyzed in this study originate from the following survey and are publicly accessible via the following repository: https://zenodo.org/records/8254376.
