# Supplementary material for: AI-supported qualitative analysis of free-text responses on home care burden and support needs in Saxony
Source: Sci Rep. 2026 Apr 2;16:11223. doi: 10.1038/s41598-026-46989-7 (PMC13046719; doi:10.1038/s41598-026-46989-7)
Supplement: Supplementary file 1 — Supplementary Material 1 [file 41598_2026_46989_MOESM1_ESM.docx]

**Supplementary note on methods 1a. Prompts for the individual work steps original version.**

*1 Du erhältst eine Tabelle mit offenen, textbasierten Antworten aus einer Befragung zur häuslichen Pflege in Sachsen. Deine Aufgabe ist es, anhand der inhaltlichen/thematischen Ausrichtung der Antworten differenzierte Themenkategorien abzuleiten.

*2 Du erhältst eine Tabelle mit offenen, textbasierten Antworten aus einer Befragung zur häuslichen Pflege in Sachsen. Deine Aufgabe ist es, **jeder einzelnen Antwort genau eine der unten aufgeführten Kategorien anhand der definierten Kriterien zuzuordnen**. Dabei soll die Zuordnung zu der Kategorie erfolgen**, welche den Inhalt der Antwort am besten widerspiegelt**.

Sollte eine Antwort **inhaltlich nicht eindeutig zuordenbar sein oder keine verwertbare Information enthalten**, ordne sie bitte der Kategorie **„Keine Angabe oder inhaltlich nicht zuordenbar“** zu.

**Kategorien zur Auswahl:**

**„Psychische Belastung und emotionale Überforderung“**→ Aussagen über Erschöpfung, depressive Verstimmung, Stress, Angst oder Überforderung im Alltag.

**„Zeitliche Belastung“**→ Hinweise auf zeitliche Belastung für den pflegenden Angehörigen durch die Pflege. Aussagen wie „Ich habe kaum noch Zeit“.

**„Antragstellung oder Bürokratie“**
→ Prozess der Antragsstellung, Unklare Verfahren, unklare Einstufung Pflegegrad, lange Bearbeitungszeiten, Ablehnungen, fehlende Unterstützung, Kommunikation mit der Krankenkasse. Aussagen wie „Die Einstufung des Pflegegrades ist nicht nachvollziehbar“, „Die Kommunikation mit der Krankenkasse ist eine Katastrophe“.

**„Erfahrungen als pflegender Angehhöriger oder mit Pflegeheimen bzw. professionellen Pflegediensten“**→ persönlichen Erfahrungen als pflegender Angehöriger und Erfahrungen mit stationären Pflegeeinrichtungen oder ambulanten Pflegediensten. Dazu gehören sowohl positive als auch negative Erlebnisse sowie neutral gehaltene Berichte. Beispielsweise Kritik an Qualität, Personalmangel, Sprachbarrieren oder fehlender Empathie.

**„Finanzielle Belastung / Hohe Pflegekosten“**→ Kritik an niedrigen Leistungen, hohe Zuzahlungen, finanzielle Belastungen, zu wenig Geld.

**„Persönliche Wünsche und Konkrete Verbesserungsvorschläge“**→ individuelle Wünsche oder Bedürfnisse im Zusammenhang mit der persönlichen Pflegesituation. Zum Beispiel Wunsch nach besserer Beratung, mehr Information oder Entlastung. Aussagen wie „Ich wünsche mir, dass man besser informiert wird“, Ich möchte selbst nicht pflegen“. Konkrete Vorschläge zur strukturellen oder politischen Verbesserung des Pflegebereichs wie beispielsweise Forderungen nach gesetzlichen Reformen oder politischen Maßnahmen, Vorschläge zur Stärkung pflegender Angehöriger durch staatliche Maßnahmen, Steigerung der Attraktivität des Pflegeberufes, Maßnahmen zur Aufklärung der Öffentlichkeit. **Aussagen wie** „Verbesserung von…“, „Pflege sollte besser bezahlt werden“, „Die Politik sollte…“.

**„Angaben zur Pflegesituation (Einzelfallbeschreibung)“**→ Neutrale Beschreibung der individuellen Pflegesituation. Zum Beispiel wer in welchem Umfang gepflegt wird und wie lange schon. Entfernung zum Wohnsitz der Angehörigen. Aussagen wie „Ich pflege meine Eltern“,

**„Noch keine oder kaum Erfahrung mit Pflege“**→ Aussagen wie „bin neu“, „erste Erfahrungen“, „noch nicht viel passiert“, „muss niemanden pflegen“.

**„Anmerkungen zum Fragebogen“**→ Rückmeldungen zur Sprache, Antwortmöglichkeiten, Länge, Verständlichkeit oder Struktur des Fragebogens. Aussagen wie „Frage war unklar formuliert“, „fehlende Antwortmöglichkeiten“

**„Keine Angabe“**→ Leere oder unverständliche Texte.

**Schritt 1: Vorschau zur Validierung**

Bevor du alle Antworten kategorisierst, zeige **für jede der oben genannten Kategorien** **zwei Beispiele aus den vorliegenden Antworten**, welche deiner Meinung nach **typisch für diese Kategorie** sind. Gib für jede Kategorie folgende Struktur aus:

- **Kategorie:** [Name der Kategorie]
- **Beispielfrage:** [Textantwort aus der Umfrage, die du dieser Kategorie zuordnen würdest]
- **Case-**Nummer
- **Begründung:** [Warum passt diese Antwort in diese Kategorie?]

Warte nach dieser Vorschau auf eine Bestätigung, bevor du mit der vollständigen Kategorisierung und der Erstellung der Excel-Tabelle fortfährst.

**Schritt 2: Nach Bestätigung – Vollständige Kategorisierung**

Nach meiner Bestätigung kategorisierst du **alle Antworten**, gibst sie als **Excel-Tabelle** mit folgenden Spalten aus:

- Case-Nummer
- **Antwort** (Originaltext der Antwort)
- **Zugeordnete Kategorie** (Name der passenden Kategorie)

*3 Du erhältst eine Tabelle mit Antworten aus einer Befragung zur häuslichen Pflege in Sachsen. Fasse getrennt zusammen, welche persönlichen Wünsche und konkreten Verbesserungsvorschläge für das Pflegesystem durch die Personen genannt werden. Gib auch an, wie oft bestimmte Punkte genannt werden.

Du erhältst eine Tabelle mit Antworten aus einer Befragung zur häuslichen Pflege in Sachsen. Fasse getrennt zusammen, welche Erfahrungen von den Befragten in ihrer Rolle als pflegende Angehörige genannt werden. Gib auch an, wie oft bestimmte Punkte genannt werden.

Du erhältst eine Tabelle mit Antworten aus einer Befragung zur häuslichen Pflege in Sachsen. Fasse getrennt zusammen, welche Angaben die Befragten zur individuellen Pflegesituation gemacht haben. Gib auch an, wie oft bestimmte Punkte genannt werden.

Du erhältst eine Tabelle mit Antworten aus einer Befragung zur häuslichen Pflege in Sachsen. Fasse getrennt zusammen, welche Angaben die Befragten zur individuellen Pflegesituation gemacht haben. Gib auch an, wie oft bestimmte Punkte genannt werden.

Du erhältst eine Tabelle mit Antworten aus einer Befragung zur häuslichen Pflege in Sachsen. Fasse getrennt zusammen, welche Probleme von den Befragten in Bezug auf die Antragsstellung oder den bürokratischen Aufwand genannt werden. Gib auch an, wie oft bestimmte Punkte genannt werden.

Du erhältst eine Tabelle mit Antworten aus einer Befragung zur häuslichen Pflege in Sachsen. Fasse zusammen, welche Angaben die befragten Personen vor dem Hintergrund von finanzieller Belastung durch die Pflege oder hoher Pflegekosten gemacht haben. Gib auch an, wie oft bestimmte Punkte genannt werden.

**Supplementary note on methods 1b. Prompts for the individual work steps English translation.**

*1 You will receive a table containing open-ended, text-based responses from a survey on home care in the German state of Saxony. Your task is to identify and derive differentiated thematic categories based on the content and subject matter of the responses.

*2 You will receive a table containing open-ended, text-based responses from a survey on home care in Saxony. Your task is to assign **each individual response to exactly one of the predefined categories listed below, based on the specified criteria**. Each response should be classified under the category **that best reflects its content**. If a response cannot be **clearly assigned or does not contain any usable information**, please categorize it under: **"No information provided or not thematically classifiable.”**

**Categories:**

**“Mental stress and emotional overload”**→ Mentions of exhaustion, depressive moods, stress, anxiety, or feeling overwhelmed in everyday life.

**“Time-related burden”**→ Indications of time constraints due to caregiving. Statements like “I barely have time anymore.”

**“Application process or bureaucracy”**→ Comments on the application process, unclear procedures, vague assessment of care levels, long processing times, rejections, lack of support, or communication with health insurance. **Examples**: “The assessment of the care level is incomprehensible”, “Communication with the health insurance is a disaster.”

**”Experiences as a caregiver or with care homes / professional care services”**→ Personal experiences as a caregiver, or experiences with nursing homes or professional home care services. Includes both positive and negative experiences, as well as neutral accounts. Examples include criticism of quality, staff shortages, language barriers, or lack of empathy.

**“Financial burden / High care costs”**→ Criticism of low benefits, high co-payments, financial stress, insufficient funding.

**“Personal wishes and concrete suggestions for improvement”**→ Individual wishes or needs related to the personal caregiving situation. **Examples:** “I wish for better information”, “I don’t want to provide care myself.” Also includes concrete suggestions for structural or political improvements, such as calls for legislative reform, strengthening family caregivers through state measures, or increasing the attractiveness of care professions. **Examples:** “Improvement of…”, “Care work should be better paid”, “Politics should…”

**“Information on the individual care situation (case descriptions)”**→ Neutral descriptions of the individual care situation, such as who is being cared for, the scope of care, and for how long. **Examples:** “I care for my parents”, “I’ve been doing this for five years.”

**“Little or no experience with caregiving”**→ Statements like “I’m new to this”, “just starting”, “not much has happened yet”, “I’m not caring for anyone.”

**“Comments on the questionnaire”**→ Feedback on language, answer options, length, clarity, or structure of the questionnaire. **Examples:** “The question was unclear”, “missing answer options.”

**“No information provided”**→ Empty or unintelligible responses.

**Step 1: Preview for Validation**

Before proceeding with the full categorization, please display two typical examples from the available responses for each of the above categories, using the following format:

- **Category:** [Name of the category]
- **Example response:** [Text of the selected survey response]
- **Case** number
- **Justification:** [Explain why this response fits this category]

Wait for confirmation before continuing with the full categorization and generating the Excel table.

**Step 2: Upon Confirmation – Full Categorization**

After confirmation, categorize all responses and output them in an Excel table with the following columns:

- Case number
- **Response** (Original text)
- **Assigned category** (Name of the selected category)

*3 You will receive a table with answers from a survey on the care situation in Saxony. Summarize separately the personal wishes and specific suggestions for improvement for the care system mentioned by the respondents. Also indicate how often certain points are mentioned.

You will receive a table with answers from a survey on the care situation in Saxony. Summarize separately the experiences mentioned by the respondents in their role as family caregivers. Also indicate how often certain points are mentioned.

You will receive a table with answers from a survey on the care situation in Saxony. Summarize separately what information the respondents provided about their individual care situation. Also indicate how often certain points are mentioned.

You will receive a table with answers from a survey on the care situation in Saxony. Summarize separately what information the respondents provided about their individual care situation. Also indicate how often certain points are mentioned.

You will receive a table with answers from a survey on the care situation in Saxony. Summarize separately the problems mentioned by the respondents in relation to the application process or the bureaucratic effort involved. Also indicate how often certain points are mentioned.

You will receive a table with answers from a survey on the care situation in Saxony. Summarize the information provided by the respondents regarding the financial burden of care or high care costs. Also indicate how often certain points are mentioned.
