# Supplementary material for: AI-supported qualitative analysis of free-text responses on home care burden and support needs in Saxony
Source: Sci Rep. 2026 Apr 2;16:11223. doi: 10.1038/s41598-026-46989-7 (PMC13046719; doi:10.1038/s41598-026-46989-7)
Supplement: Supplementary file 2 — Supplementary Material 2 [file 41598_2026_46989_MOESM2_ESM.docx]

Supplementary Table S1. Frequency distribution of mentions by subcategory.

| **Main Category** | **Subcategories** | **Number of mentions (n)** | **Number of mentions (%)** |
| --- | --- | --- | --- |
| Personal wishes and specific suggestions for improvement | **Personal wishes** |  |  |
|  | Care at home instead of in a nursing home, remaining independent for as long as possible | 15 | 4.5 |
|  | No burden on children/family due to care | 6 | 1.8 |
|  | More social contact, less isolation | 4 | 1.2 |
|  | **Specific suggestions for improvement** |  |  |
|  | Better pay and more staff in nursing care | 20 | 6.0 |
|  | Desire for more relief options | 8 | 2.4 |
|  | Improve the financial situation of home care | 16 | 4.8 |
|  | Reduce bureaucracy, provide unbureaucratic assistance | 9 | 2.7 |
|  | Improve language skills among foreign nursing staff | 4 | 1.2 |
|  | Care should not be profit-oriented, but organized by the state | 7 | 2.1 |
|  | Adjustment of long-term care insurance (e.g., inclusion of civil servants) | 8 | 2.4 |
|  | More flexible and needs-based benefits in kind/care services | 4 | 1.2 |
|  | More state control/transparency of nursing homes | 5 | 1.5 |
|  | Expansion of alternative forms of living (e.g., shared apartments, multi-generational housing) | 6 | 1.8 |
|  | Better transitions from hospital to care | 3 | 0.9 |
|  | Better care in rural areas | 4 | 1.2 |
|  | Promotion of volunteer help and social engagement | 3 | 0.9 |
|  | Improved information about care levels, rights, applications, etc. | 6 | 1.8 |
|  | Better compatibility of care and work | 5 | 1.5 |
|  | Promotion of palliative care, more hospices | 3 | 0.9 |
| Application process | High bureaucratic effort | 10 | 3.0 |
|  | Incorrect classification by medical services | 6 | 1.8 |
|  | Long processing times/delays in recognition | 5 | 1.5 |
|  | Lack of transparency regarding responsibilities and services | 5 | 1.5 |
|  | Applications are rejected or only approved with considerable effort | 5 | 1.5 |
|  | Incomprehensible forms | 4 | 1.2 |
|  | Unclear communication | 4 | 1.2 |
|  | Lack of automatic or timely information | 3 | 0.9 |
|  | Inappropriate behavior by employees | 2 | 0.6 |
| Financial  burden | Direct financial burden due to care costs | 6 | 1.8 |
|  | Burden due to impending income assessment or maintenance obligations | 3 | 0.9 |
|  | Inadequate support from the system / lack of relief | 4 | 1.2 |
|  | Impact on own employment / retirement provision | 4 | 1.2 |
| Experience as a informal caregiver or with nursing homes or professional care services | **Experience as a informal caregiver** |  |  |
|  | High stress / exhaustion | 15 | 4.5 |
|  | Lack of support / relief | 11 | 3.3 |
|  | Insufficient information and advice | 9 | 2.7 |
|  | Negative experiences with health insurance companies / medical services | 4 | 1.2 |
|  | **Experience with nursing homes/outpatient care services (subcategories)** |  |  |
|  | Staff shortages/time pressure | 18 | 5.4 |
|  | Costs and financing problems | 13 | 3.9 |
|  | Quality of care varies greatly | 10 | 3.0 |
|  | Impersonal and passive home environment | 8 | 2.4 |
|  | Grievances | 5 | 1.5 |
|  | Home care in combination with outpatient services rated positively | 7 | 2.1 |
|  | Bureaucracy overburdens care services | 4 | 1.2 |
| Information on the care situation (description of individual case) | **Care of relatives (current or completed)** |  |  |
|  | Care of parents | 20 | 6.0 |
|  | Care of husband/wife | 8 | 2.4 |
|  | Care of child | 5 | 1.5 |
|  | Care of parents-in-law | 4 | 1.2 |
|  | Care of friends/extended family | 3 | 0.9 |
|  | **Own need for care** |  |  |
|  | In need of care with care level | 10 | 3.0 |
|  | Serious illness without care level | 6 | 1.8 |
|  | **Deceased person in need of care** |  |  |
|  | Care ended due to death | 15 | 4.5 |

Note: Percentages refer to the total number of free-text responses (N = 332).
