# Supplementary material for: AI-supported qualitative analysis of free-text responses on home care burden and support needs in Saxony
Source: Sci Rep. 2026 Apr 2;16:11223. doi: 10.1038/s41598-026-46989-7 (PMC13046719; doi:10.1038/s41598-026-46989-7)
Supplement: Supplementary file 3 — Supplementary Material 3 [file 41598_2026_46989_MOESM3_ESM.docx]

Supplementary Table S2. Overview of error types identified in AI-based text categorization of free-text responses.

| **Error Type** | **Description** | **Example (English translation)** | **Frequencies (n)** | **Frequencies (%)** |
| --- | --- | --- | --- | --- |
| Semantic Overlap | Similar terms or themes led to incorrect categorization. | “As a caregiver, there are no short-term or hourly replacement options to get some relief.” → incorrectly classified as time-related burden instead of Experience as a informal caregiver or with nursing homes or professional care services. | 16 | 10.4 |
| Ambiguity/ Vagueness | Unclear or very brief statements lacking sufficient context for classification. | 1. We donate regularly: SOS Children's Villages Mouth and hand painting artists Spontaneously: War refugees 2. Currently in the midst of moving -> new apartment without any stairs | 10 | 6.5 |
| Unusual Expressions | Idiomatic or regionally specific phrases were not correctly interpreted. | These different services are a nightmare; it's impossible to understand them, they're complicated and incomprehensible. | 7 | 4.5 |
| Context Misinterpretation | The model failed to correctly interpret the situational or referential context of the statement, leading to an incorrect thematic assignment. | In the selection field for occupation, self-employment is missing.” → incorrectly classified as no information or not assignable instead of comments on the questionnaire. | 84 | 54.5 |
| Formatting or Punctuation Misreading | Lack of punctuation or unconventional formatting led to incorrect segmentation or misclassification. | [FB 311, 10 pages of handwritten annex] We request assistance from your organization. [...] | 4 | 2.6 |
| Lack of Context Consideration | The statement contained multiple aspects, but the model selected only one. | Care level 1. One hour every two weeks is not enough. Changing staff is time-consuming and stressful for the person receiving care.  (Assigned only to the category “Information on the care situation (individual case description)” 🡪This response also fit the category “Experiences as a family caregiver or with professional care services.” | 33 | 21.4 |

Note. The numbers refer to all incorrectly assigned responses, including cases in which only one category (n=121)was assigned although multiple categories would have been applicable (n=33).
