# Supplementary figures and images for: AI-supported qualitative analysis of free-text responses on home care burden and support needs in Saxony
Source: Sci Rep. 2026 Apr 2;16:11223. doi: 10.1038/s41598-026-46989-7 (PMC13046719; doi:10.1038/s41598-026-46989-7)

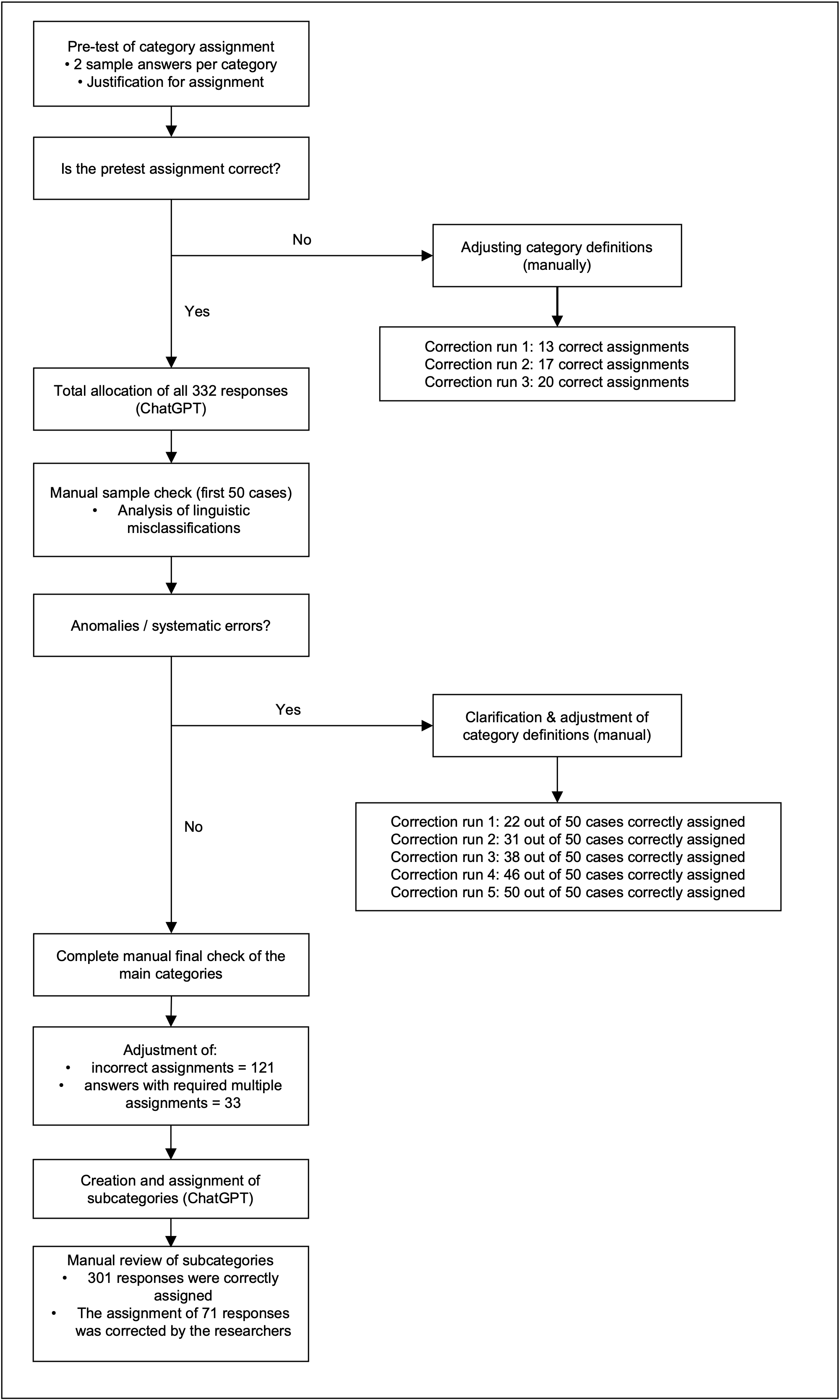

Supplement: Supplementary file 4 — Supplementary Material 4 [file 41598_2026_46989_MOESM4_ESM.png]
